# Rapid Detection and Identification of Human Hookworm Infections through High Resolution Melting (HRM) Analysis

**DOI:** 10.1371/journal.pone.0041996

**Published:** 2012-07-26

**Authors:** Romano Ngui, Yvonne A. L. Lim, Kek Heng Chua

**Affiliations:** 1 Department of Parasitology, Faculty of Medicine, University of Malaya, Kuala Lumpur, Malaysia; 2 Department of Molecular Medicine, Faculty of Medicine, University of Malaya, Kuala Lumpur, Malaysia; The George Washington University Medical Center, United States of America

## Abstract

**Background:**

Hookworm infections are still endemic in low and middle income tropical countries with greater impact on the socioeconomic and public health of the bottom billion of the world's poorest people. In this study, a real-time polymerase chain reaction (PCR) coupled with high resolution melting-curve (HRM) analysis was evaluated for an accurate, rapid and sensitive tool for species identification focusing on the five human hookworm species.

**Methods:**

Real-time PCR coupled with HRM analysis targeting the second internal transcribed spacer (ITS-2) of nuclear ribosomal DNA as the genetic marker was used to identify and distinguish hookworm species in human samples. Unique and distinct characteristics of HRM patterns were produced for each of the five hookworm species. The melting curves were characterized by peaks of 79.24±0.05°C and 83.00±0.04°C for *Necator americanus*, 79.12±0.10°C for *Ancylostoma duodenale*, 79.40±0.10°C for *Ancylostoma ceylanicum*, 79.63±0.05°C for *Ancylostoma caninum* and 79.70±0.14°C for *Ancylostoma braziliense*. An evaluation of the method's sensitivity and specificity revealed that this assay was able to detect as low as 0.01 ng/µl hookworm DNA and amplification was only recorded for hookworm positive samples.

**Conclusion:**

The HRM assay developed in this study is a rapid and straightforward method for the diagnosis, identification and discrimination of five human hookworms. This assay is simple compared to other probe-based genotyping methods as it does not require multiplexing, DNA sequencing or post-PCR processing. Therefore, this method offers a new alternative for rapid detection of human hookworm species.

## Introduction

Hookworms are blood feeding intestinal nematodes that infect almost 600 million people worldwide, resulting in up to 135,000 deaths annually [1]. *Necator americanus* and *Ancylostoma duodenale* are two most common species causing infection in humans. In general, mixed infections of these hookworms are common in many endemic areas especially among people in tropical and subtropical countries with low socioeconomic status. Besides the two human species, canine and/or feline hookworms such as *Ancylostoma ceylanicum*, *Ancylostoma caninum* and *Ancylostoma braziliense* can also cause infections to human. Recently, zoonotic ancylostomiasis caused by *A. ceylanicum* has been occasionally reported in rural communities in Malaysia [2], Thailand [3] and Laos PDR [4]. The gravest consequences are manifested in children and women of childbearing age [5] displaying chronic intestinal blood loss which may result in iron-deficiency, anaemia and hypoalbuminemia [6], [7]. The most deleterious effects of hookworm infections include impaired physical, intellectual and cognitive development of children, increased mortality in pregnant women and their infants and reduced work capacity of adolescents and adults [5]–[9].

Accurate diagnosis and genetic characterization of hookworms are essential for the formulation of effective control measures. Currently, most research conducted on the epidemiology of hookworm and other intestinal nematodes has relied on the use of conventional microscopy for the identification of eggs in faeces and third-stage larvae (L3) through the coproculture technique. The benefits of this method are mainly due to technical simplicity and low cost. However, utilization of microscopy is limited by the fact that most of the nematode eggs are morphologically indistinguishable from those of other species, and it is laborious, time-consuming and requires relatively skilled personnel. Thus, there is a crucial need for a practical, highly sensitive and specific diagnostic and analytical tool, particularly one based on the polymerase chain reaction (PCR) [10] to address key epidemiology and population genetic questions to underpin surveillance, treatment and control programme.

Following extensive evaluation of the specificity of genetic markers of hookworm such as first (ITS-1) and second (ITS-2) internal transcribed spacer of nuclear ribosomal DNA (rDNA), several techniques have been developed for the identification and characterization of hookworm at the molecular level [10]. These include conventional and semi-nested PCR [11] and single-strand conformation polymorphism (SSCP) [12], mutation scanning [13] and PCR-restriction fragment length polymorphism (RFLP) [14]. Although these approaches are very useful and effective, the electrophoretic analysis can be quite time consuming to perform. Moreover, the amplification and detection of DNA are prone to contamination and are expensive, with the endpoint reading on agarose gels yielding no quantitative information. Additionally, multiplex real-time PCR using fluorescent detection probes through the possibility of combining assays for the detection of different targets into one reaction and has been developed for the diagnosis of hookworm infection in humans [15], [16]; however, this technique is relatively expensive.

Due to the increased demand for rapid, high-throughput diagnosis and genetic analysis of pathogens as well as data handling and analysis, there has been a considerable focus on the evaluation and development of advanced detection methods which obviate the need for electrophoretic analysis, reduce the risk of contamination and substantially decrease labour time and reagent costs. High-resolution melting (HRM) analysis is a relatively new post-PCR analysis that allows direct characterization of PCR amplicons in a closed system. Probe-free HRM real-time PCR does not require the multiplex method, has no manual post-PCR processing, is performed in a closed-tube system and has a low reaction cost relative to other methods for rapid screening and detection of closely related species in a laboratory.

To date, the HRM has mostly been used in human clinical studies [17]–[21]. However, the application of the HRM technique to the diagnosis of parasitic organisms has been rather limited and the method has mostly been applied in molecular studies of parasitic protozoa such as the old world *Leishmania* spp. [22], *Cryptosporidium* spp. [23], *Plasmodium falciparum* [24], *Dientamoeba fragilis* [25], *Naegleria* spp. [26] and *Giardia* spp. [27]. As for parasitic worms, the application of the HRM method has been rather sporadic. The technique has been used for rapid discrimination of *Brugia malayi* and *Brugia pahangi* [28] and population studies of *Fascioloides magna* [29]. In the present study, we describe a new application of HRM employing the ITS-2 of nuclear ribosomal DNA as the genetic marker for the rapid detection, quantification and speciation of hookworm species in human samples. The distinction in melting curve data could be practical and beneficial for dynamic surveys and epidemiological studies of the parasite. In addition, the specificity and sensitivity of this assay in comparison with microscopy and conventional PCR are discussed.

## Materials and Methods

### Control

To establish the PCR assay, a well-defined hookworm genomic DNA was obtained from faecal samples in which *Necator americanus* (n = 10), *A. ceylanicum* (n = 10) and *A. caninum* (n = 10) were confirmed by the PCR coprodiagnostic technique, as described previously [2]. Additionally, due to limited positive controls for other species, genomic DNA of *A. duodenale* (n = 5) and *A. braziliense* (n = 5) were isolated from individual adult worms (kindly supplied by Dr. Megumi Sato from Niigata University, Japan). Genomic DNA was extracted from individual adult worms using the QIAamp DNA Mini Kit (QIAgen, Hilden, Germany) according to the manufacturer's instructions. Briefly, individual worms were suspended in 180 ATL tissue lysis buffer (QIAgen, Hilden, Germany) before being treated with sodium dodecyl sulfate-proteinase K followed by incubation at 56°C for 1 hour. Extracted DNA was then stored at −20°C until required for PCR amplification.

The assay's specificity was evaluated using a set of control DNA standards including DNA from a faecal sample of an individual with no history of parasitic infections and DNA from other parasitic nematodes (i.e., *Ascaris lumbricoides*, *Trichuris trichiura*, *Strongyloides stercoralis* and *Trichostrongylus* spp.) and protozoa (i.e., *Giardia lamblia*, *Cryptosporidium* spp., *Blastocystis* spp., *Entamoeba histolytica*, *Entamoeba dispar* and *Entamoeba moshkovskii*). Prior to PCR amplification, genomic control DNA for the different types of intestinal parasites and also human faecal samples infected with hookworm were extracted using the PowerSoil DNA Kit (MO BIO, cat. no. 12888-100, CA, USA) according to the manufacturer's instructions. Briefly, approximately 0.2 to 0.3 g of faecal sample were added into the PowerBead Tube, followed by incubation at 70°C for 10 minutes in the presence of cell lysis and disruption agent provided in the kit. Subsequently, the faecal samples were subjected to homogenization and lysis procedures for complete cell lysis by mechanical shaking (vortexing) using the MO BIO Vortex Adapter (MO BIO, cat. no. 13000-V1). All the control DNA samples were subjected to the same amplification procedure. Since the number of parasite genomes present in each sample cannot be accurately determined, the lowest detectable concentration, i.e., sensitivity of the assay, was assessed using 10-fold serial dilutions of 10 ng/µl positive control of the genomic DNA, ranging from 10^−1^ to 10^−5^. DNA concentrations were qualitatively measured using a NanoPhotometer (IMPLEN, Germany).

### Pre-amplification

Approximately 180–200 bp within the 5.8S and second internal transcribed spacer (ITS-2) region of the hookworm ribosomal RNA was amplified by real-time PCR using a pair of degenerate primers UMF (Forward: 5′-CACTGTTTGTCGAACGGYAC-3′) and UMR (Reverse: 5′-AGTCSVKRRRCGATTMARCAG-3′) and then subsequently examined by HRM analysis. The primers were designed specifically to amplify all five hookworm species (i.e., *N. americanus*, *A. duodenale*, *A. ceylanicum*, *A. caninum* and *A. braziliense*) from previously published sequences in GenBank (accession numbers AF217891, EU344797, DQ438080, EU159416 and DQ438064). Briefly, the published sequences of the five hookworm species were manually aligned and edited to obtain the consensus sequence using the BioEdit Sequence Alignment version 7.0.9 program [30]. Single pair primers were designed separately with the aide of sequence analysis and Primer Express software (Applied Biosystems, Inc., CA, USA), followed by *in silico* PCR analysis as described previously [31]–[33] to ensure the designed primers were targeting to the genomic region of interest before forming the desired degenerate primers.

For preliminary optimization of the primers, a series of gradient PCR assays using conventional PCR was carried out using a wide range of isolated DNA, i.e., non-infected humans, hookworms and DNA from other intestinal parasites, in order to obtain the optimal annealing temperature for the primers. Briefly, the PCR was carried out using 50 µl of PCR mixture containing 10× PCR buffer, 1.25 mM dNTPs, 4 mM MgCl_2_, 10 pmol of each primer, 1 U of *Taq* polymerase and 6 µl of DNA template. The sample was heated at 94°C for 5 min, followed by 30 cycles of 94°C for 30 s (denaturing), 50°C to 60°C for 30 s (gradient annealing temperature), 72°C for 30 s (extension) and a final extension at 72°C for 7 min. DNA blank and positive genomic DNA were also included during each PCR optimization.

### HRM-real-time PCR assay

Upon completion of the primer optimization using conventional PCR, real-time PCR was performed in a total reaction mixture of 20 µl containing 10 µl of MeltDoctor HRM Master Mix (Applied Biosystems, Inc., CA, USA), 10 pmole of each primer, approximately 10 ng/µl of genomic DNA and sterile deionized water using a 7500 Fast real-time PCR system (Applied Biosystems, Inc., CA, USA). Genomic DNA of positive controls (hookworm) and control samples without DNA (DNase free water, Sigma Cat. no. W4502) were included in each PCR run. The PCR thermocycling conditions was set according to the optimized protocol at 95°C for 10 min (1 cycle) followed by amplification for 40 cycles consisting of 95°C for 15 sec (denaturation step) and 60°C for 1 min (annealing and elongation steps).

Following the real-time PCR, amplicon dissociation was immediately started by a melting step in the same real-time PCR machine. The program consisted of denaturation at 95°C for 10 sec, 57°C for 1 min (annealing), 95°C for 15 sec (high resolution melting) and final annealing at 60°C for 1 min. In this process, the PCR amplicons were allowed to denature and re-anneal before the high resolution melting recording changes in fluorescence with changes in temperature (d*F*/d*T*) and plotting against changes in temperature. The high resolution melting curve profile was then analyzed using HRM analysis software version 2.0.1 with fluorescence (melting curve) normalization by selecting the linear region before and after the melting transition. Melting temperature (T_m_) was interpolated from the normalized data as the temperature at 50% fluorescence. Different genotypes were easily distinguished by plotting the fluorescence difference between normalized melting curves. All samples of hookworm species were examined in triplicate to obtain the standard deviation (SD) for the melting temperature (T_m_).

### DNA sequencing

Before this approach was used for the screening of all studied samples, confirmation of the five hookworm species which served as positive controls was done on the basis of a homology search using the Basic Local Alignment Search Tool (BLAST) program hosted by NCBI, i.e., National Centre for Biotechnology Information reference sequences (http://www.ncbi.nlm.nih.gov). Briefly, two randomly selected positive amplicons of each species derived from PCR-HRM that displayed distinct curve shapes and Tm were purified using the QIAquick Gel Extraction Kit (QIAgen, cat. no. 28104, Hilden, Germany), according to the manufacturer's instructions. The samples were then subjected to DNA sequencing in both directions (forward and reverse primers) with an ABI 3730XL sequencer (Bioneer Corporation, South Korea).

### Comparison between HRM-real-time PCR assay and conventional semi-nested PCR

The performance, i.e., sensitivity and specificity, of the real-time PCR-HRM assay for the detection of hookworm infection was determined by assaying 634 samples. The results obtained were compared to iodine stained direct smear and conventional semi-nested PCR as reported previously [2], [34]. The study protocol (MEC Ref. No. 824.11) was approved by the Ethics Committee of the University Malaya Medical Centre (UMMC), Malaysia. Briefly, consent was taken either in written form (signed) or verbally followed by thumbprints (for those who were illiterate) from the participants or their parents/guardians (on behalf of their children). For very old participants or incompetent adults, the questionnaire was completed by interviewing the relevant family member (normally head of the family) who signed the informed consent form. Details of the consent approval and ethical considerations have been presented elsewhere [2].

## Results

### Specificity and sensitivity of the primer, PCR condition and amplicon

The specificity of the primer was examined using various genomic DNA from human faeces (i.e., negative for parasitic infection) and samples positive with a range of intestinal parasites (i.e., both intestinal helminth and protozoa) prior to microscopy and conventional PCR examination. In this assay, only the amplification and HRM plots of positive controls, i.e., hookworm DNA, was detected while no amplification of other genomic DNA, i.e., in human faeces negative for parasitic infections or samples of DNA from protozoa or other helminths, was observed.

Subsequently, the amplicons derived from selected genomic samples representing hookworm DNA (positive control) and other DNA samples were verified by 2% (w/v) gel electrophoresis. On agarose gels, only the amplicon from hookworm DNA (approximately 180–200 bp) was observed while no bands were detected for other parasite DNA samples. The amplicons from randomly selected samples representing all five hookworm species were sequenced for species conformation based on sequence comparison using BLAST with reference sequences from GenBank shown to represent *N. americanus* (accession number JF960390), *A. duodenale* (accession number EU344797), *A. ceylanicum* (accession number JN120876), *A. caninum* (accession number JN120895) and *A. braziliense* (accession number JF120898), respectively.

Given that the copy number of parasite genomic DNA present in each sample cannot be accurately determined, the sensitivity was assessed by using a well-defined reference DNA control to determine the lowest detectable DNA concentration in this assay. The assay sensitivity was assessed by using 10-fold serial dilutions of 10 ng/µl of predefined hookworm genomic DNA, i.e., ranging from 10^−1^ to 10^−5^. No amplification was noted at the dilution of 10^−4^ and 10^−5^, and therefore 10^−3^ (0.01 ng/µl) marked the lowest dilution at which parasite DNA was detected.

### Sample categorization based on HRM curve profile

In order to examine the reproducibility, i.e., consistency, of each melting profile, amplicons representing the reference control DNA from each hookworm species were tested in triplicate and repeated on several different days by keeping the same chemistry environment with similar reagents and DNA concentrations. Our results demonstrated that the reproducibility of the assay was very high with consistent melting patterns between runs for each species analyzed on different days.

**Figure 1:**
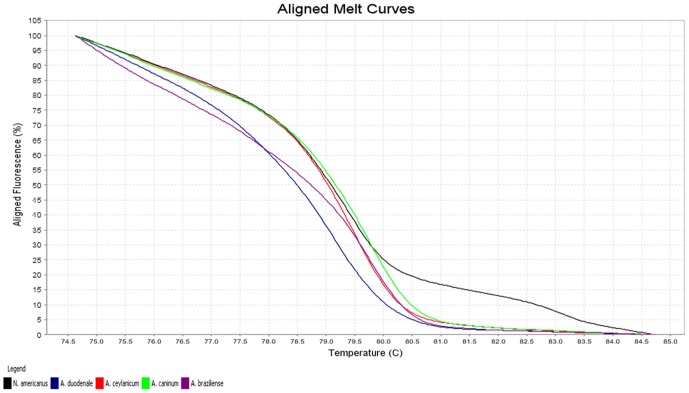
Representative profiles of the melting curves (aligned melt curves) of ITS-2 amplicons for *Necator americanus* (black), *Ancylostoma duodenale* (blue), *A. ceylanicum* (red), *A. caninum* (green) and *A. braziliense* (purple). Fluorescence is plotted against degrees Celsius (°C).

**Figure 2:**
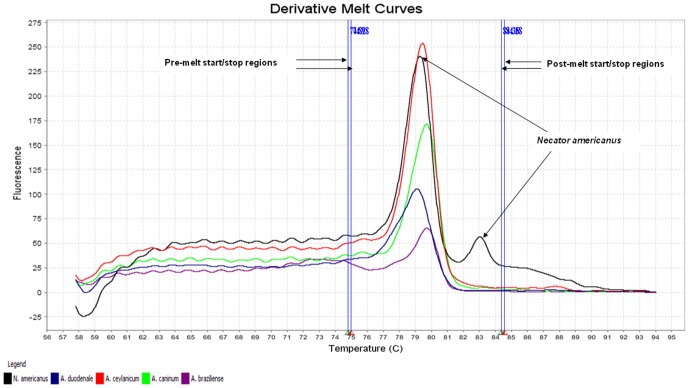
Representative profiles of the melting curves (derivative melt curves) of ITS-2 amplicons for *Necator americanus* (black), *Ancylostoma duodenale* (blue), *A. ceylanicum* (red), *A. caninum* (green) and *A. braziliense* (purple). *N. americanus* (black) produced two peaks while single peak was produced for other *Ancylostoma* spp. Pre-melt region: The set of lines to the left of the peak indicates the pre-melt start and stop temperatures when every amplicon is double-stranded. Post-melt region: The set of lines to the right of the peak indicates the post-melt start and stop temperatures when every amplicon is single-stranded.

**Figure 3:**
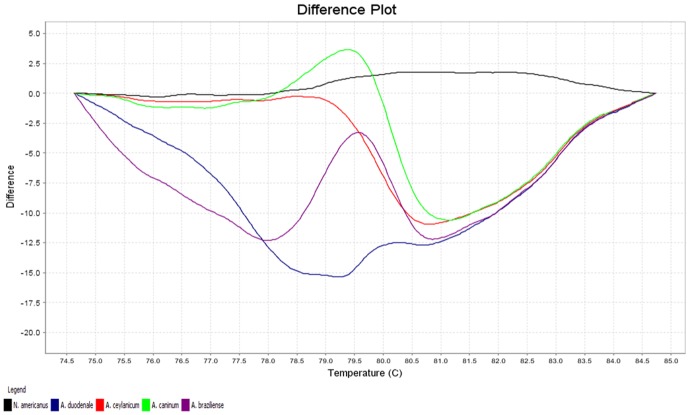
Representative profiles of the melting curves (difference plot curves) of ITS-2 amplicons for *Necator americanus* (black), *Ancylostoma duodenale* (blue), *A. ceylanicum* (red), *A. caninum* (green) and *A. braziliense* (purple).

The melting characteristics of ITS-2 amplicons from all species were assessed by plotting three different curves (Figures 1, 2, 3). In the present study, the normalized fluorescence curves, i.e., aligned melt curve (Figure 1), derivative melt curve (Figure 2) and difference plot melt curve (Figure 3) produced uniquely different plots that were easily distinguishable for each species. The melting curves were characterized by peaks of 79.24±0.05°C and 83.00±0.04°C in profile 1 (*N. americanus*), 79.12±0.10°C in profile 2 (*A. duodenale*), 79.40±0.10°C in profile 3 (*A. ceylanicum*), 79.63±0.05°C in profile 4 (*A. caninum*) and 79.70±0.14°C in profile 5 (*A. braziliense*) (Table 1). As for *Ancylostoma* spp., although their melting profiles (Tm) were almost similar to each other, they could clearly be discerned by the plotting of normalized melting curves (Figure 1) and temperature-shifted fluorescence difference (Figure 3).

**Table 1:** Results achieved by real-time PCR coupled HRM analysis of ITS-2 amplicon from control genomic DNA for hookworm.

		Mean melting temperature ± standard	
Melting curve analysis	Number of control	deviation (SD)	
	samples examined	Peak 1 (T_m_1)	Peak 2 (T_m_2)
Profile 1 (*N. americanus*)	10	79.24±0.05	83.00±0.04
Profile 2 (*A. duodenale*)	10	79.12±0.10	-
Profile 3 (*A. ceylanicum*)	10	79.40±0.10	-
Profile 4 (*A. caninum*)	5	79.63±0.05	-
Profile 5 (*A. braziliense*)	5	79.70±0.14	-

For each of the hookworm species, a sharp decreased in fluorescence was detected in denatured DNA as shown in normalized fluorescence curves (Figure 1), which was consistent with its respective melting profile (Figure 2). It was also noted that this assay easily distinguished between *N. americanus* (profile 1) and *Ancylostoma* spp. (profiles 2 to 5) by the presence of two peaks in profile 1 compared to a single peak in the other profiles representing *Ancylostoma* spp. as demonstrated in the derivative melt curve (Figure 2).

### Comparison between HRM-real-time PCR assay and conventional semi-nested PCR

The comparison between microscopy, conventional semi-nested PCR and the real-time PCR-HRM assay was also explored (Table 2). Fifty-eight out of 634 faecal samples were microscopically positive for hookworm-like eggs. However, specific hookworm amplification was only detected in 47 (81.0% of 58) samples via conventional semi-nested PCR in which hookworm-like eggs were seen in the iodine stained direct smear examination. The same faecal samples (N = 634), regardless of the infection status, were also analyzed for species identification by HRM real-time PCR. Species identified in all positive samples were in accordance to our previously published work [2], [34].

**Table 2:** Comparison between microscopy, conventional semi-nested PCR and real-time PCR-HRM assays (N = 634).

		Semi-nested PCR^a^^; ^ b				Real-time PCR-HRM^c^			
Microscopy^a^		Positive		Negative		Positive		Negative	
	n	n	%	n	%	n	%	n	%
Positive	58	47	81.0	11	19.0	58	100	0	0
Negative	576	0	0	576	100	0	0	576	100

a Details have been published elsewhere [2], [34].

b Sensitivity: 84.1%; Specificity: 100% (Figure S1).

c Sensitivity: 100%; Specificity: 100% (Figure S1).

In our real-time PCR-HRM assay, specific amplification was detected in all 58 (100%) samples in which hookworm-like eggs were seen by microscopy. No amplification was detected in 576 microscopically negative samples by both the conventional semi-nested PCR and the HRM real-time PCR assays leaving the true prevalence of the hookworm infections in the studied population as 10.1% (58 of 634).

The sensitivity and the specificity of the conventional semi-nested PCR and HRM-real-time PCR assays for detection of hookworm infections were also evaluated in the present study. As for the sensitivity (i.e., the ability of the assay to identify true positive hookworm infections), both assays gave 100% sensitivity (Figure S1). With regards to the specificity (i.e., the ability of the assay to identify true negative hookworm infections), HRM real-time PCR assay (100%) had higher specificity as compared to conventional semi-nested PCR (84.1%).

In our previous work, we were not able to amplify 11 samples in which hookworm-like eggs were seen via microscopy; however, these samples were amplified and identified as *N. americanus* based on their melting profile by the HRM assay (Figure S2). In addition, five mixed infections of *N. americanus* and *A. ceylanicum* detected in our previous work also produced a unique melting point plot that was easily distinguishable from single infection cases via our HRM real-time PCR assay (Table 3).

**Table 3:** Hookworm species detected via both conventional semi-nested PCR and real-time PCR-HRM assays (N = 58).

Hookworm species	Semi-nested		Real-time	
	PCR^a^		PCR-HRM	
*Necator americanus*	36	62.1	47	81.1
*Ancylostoma ceylanicum*	6	10.3	6	10.3
Mixed (*N. americanus and A. ceylanicum*)	5	8.6	5	8.6
Negative	11	19.0	0	0
Total	58	100	58	100

a Details have been published elsewhere [2], [34].

## Discussion

It is known that the majority of epidemiology studies of parasitic worm infections, including hookworm species, rely mainly on conventional microscopy as the diagnostic gold standard. Although this method is technically simple and not costly, it is hampered by the fact that most nematode parasites, especially from the Strongiloidae family, are morphologically indistinguishable from each other. Besides that, the technique is laborious to perform, time-consuming and requires skilled personnel. In recent years, several molecular techniques, mainly those based on PCR have been developed for the specific identification and characterization of hookworm infection [11]–[16]. Although these techniques are sensitive and specific for the identification of hookworm species, they are laborious and time consuming, especially the post-PCR processing steps. In addition, there is also a higher risk of contamination, they are more expensive (e.g., DNA sequencing) and the techniques only provide qualitative information.

In the present study, we have successfully utilized HRM analysis coupled with real-time PCR for the rapid detection, quantification and species identification of hookworm species in human samples. HRM analysis is a completely “closed tube”, probe-based genotyping assay that does not employ additional post-PCR steps and simply utilizes a DNA melting assay and computerized analysis to produce graphic output, thus lowing the risk of contamination [35], [36]. It measures changes in the rate of double stranded DNA (dsDNA) dissociation to single stranded DNA with increasing temperature. HRM analysis starts with PCR amplification of the region of interest in the presence of a dsDNA-binding dye. This binding dye has high fluorescence when bound to dsDNA and low fluorescence when in the unbound state. When the dsDNA dissociates (melts) into single stranded DNA, the dye is released, causing a change in fluorescence. Amplification is followed by a high-resolution melting step. The observed melting behaviour is characteristic of the particular DNA products as determined based on their composition, length, GC content, complementarity and nearest neighbour thermodynamics [35], [36].

To the best of our knowledge, this is the first report on the utilization of the HRM approach for rapid detection and discrimination of nematode, i.e., hookworm, infection by employing the ITS-2 of nuclear ribosomal DNA as a genetic marker. Since the first introduction of HRM analysis in 2003 [17], it has been widely applied in clinical studies such as in mutation scanning [17], genotyping of single base changes [18], sequence matching [19], insertions or deletions [20] and detection of single nucleotide polymorphisms (SNPs) [21]. As for parasitic organisms, it has been used sporadically and mainly for the study of parasitic protozoa such as for rapid detection of point mutations associated with antimalarial drug resistance in *Plasmodium falciparum* genes [24]. Similarly, this technique has been applied for the differentiation of Old World *Leishmania* spp. in both human and animal samples [22]. Robinson et al. [26] used melting curve analysis of ITS to distinguish the various *Naegleria* species. Similarly, Pangasa et al. [23] applied the ITS-2 spacer for the rapid screening of several *Cryptosporidium* spp., while the small ribosomal subunit was used a tool in the HRM analysis of the genetic diversity of different clinical isolates of *Dientamoeba fragilis* [25]. Additionally, a similar technique has been used in parasitic worm studies such as for a rapid identification of the two closely related filariasis worms, i.e., *Brugia malayi* and *Brugia pahangi* [28]. More recently, similar method has also been developed for effective population studies of *Fascioloides magna* [29].

The present study has shown that HRM can be used to easily distinguish among various hookworm species based on the distinctive characteristics of the repeatable curves and melting temperatures for each species although samples were obtained from different hosts (i.e., human vs animals), sources (i.e., faeces vs adult worm) and life cycle stages (i.e., eggs vs adult worm). The results revealed that similar melting curves and profiles were generated regardless of whether the sources of the genomic DNA were derived from adult worms or eggs found in human or animal faeces. This finding was in accordance with a recent study on the identification of Old World *Leishmania* using a similar approach in which the melting curve was reproducible despite the fact that the *Leishmania* strains originated from different locations, hosts (i.e., human vs reservoir host) and vectors (i.e., sand flies) [22].

In this assay, discrimination between the different genera of *N. americanus* and *Ancylostoma* spp. was straightforward as shown by the presence of double-peaks for *N. americanus* but only a single peak for *Ancylostoma* spp. Similar findings were observed in the study of Old World *Leishmania* in which distinctly different curves were produced for non-leishmanial trypanosdomatids and *Leishmania* spp. [22]. Additionally, the appearance of multi-peaks for *N. americanus* was reproducible and has been demonstrated in various HRM analysis studies [35]–[38]. The presence of multi-peaks can be used as an additional diagnostic criterion for species or genotype discrimination. More recently, a study conducted to differentiate *Cryptosporidium* species using HRM analysis also found multi-peaks for *C. hominis* but not for *C. parvum* or *C. meleagridis* [23]. In general, different genotypes have their own unique transitions that are shown by their HRM profile, shape comparison and difference plots of their melting curves [17]. In the case of double peaks, the lower T_m_s peaks were always smaller than the higher peaks, showing the heteroduplex genotype of the melting transition while samples with a single peak indicated a homozygous genotype [17].

The HRM analysis reported here also revealed that the approach has the capability to detect ‘mixed’ infections in which uniquely distinct melting curves were produced from previously classified genomic DNA positive with *N. amercianus* and *A. ceylanicum* as reported in our previous studies. A number of previous studies have reported mixed hookworm infections in humans via utilization of conventional methods such as DNA sequencing and RFLP analysis. For instance, a recent study conducted in Thailand revealed that a participant was harboring a mixed infection of *N. americanus* and *A. ceylanicum* as detected by direct DNA sequencing [3]. Similarly, mixed infections of *N. americanus* and *A. duodenale* were also reported in Lao PDR [4] and Ghana [39]. This finding is in keeping with a study conducted on the diagnosis of human cryptosporidiosis using a similar HRM analysis, in which the assay could detect mixed infections of *C. hominis* and *C. parvum* prior to SSCP analysis [23]. However, Pangasa et al. [23] also noted that the ability of HRM analysis to detect mixed infection is not expected to achieve consistent sensitivity and accuracy compared to other probe-based genotyping methods such as SSCP. Thus, this limitation can be overcome in future study by combining the current HRM assay with other assays such as multiplex-tandem PCR [40] or probe-PCR [41].

The current HRM assay is more sensitive and specific for the detection and discrimination of hookworm species compared to the conventional semi-nested PCR, as evidenced by the achievement of 100% sensitivity and specificity for the detection of *N. americanus* and *A. ceylanicum* (previously confirmed based on DNA sequencing data). In our previous work, there were 11 samples in which hookworm-like eggs were observed via microscopy but failed to be amplified through conventional PCR. These were identified as *N. americanus* based on the melting profile in the HRM assay in this study. All microscopy negative samples were also subjected to HRM real-time PCR to make sure that they were not misdiagnosed, i.e., false negative cases of hookworm infection. The results were in accordance with our previous work where none of the microscopically negative sample were amplified via the HRM assay. Additionally, the ability of the current HRM assay to detect as low as 0.01 ng/µl DNA and its inability to produce any amplification of control DNA representing a wide range of non-hookworm intestinal nematodes and protozoa indicated its potential as an alternative diagnostic tool to other probe-based genotyping assays.

In conclusion, the current real-time PCR assay coupled with HRM analysis can serve as an alternative molecular epidemiology tool for rapid screening of large numbers of samples. The advantages over other methods used for species differentiation and discrimination include its rapidness, simplicity as no electrophoresis is required to verify the product sustainably reducing processing time, as well as its high specificity and sensitivity. Moreover, HRM analysis does not require a special instrument as it can be performed using an existing real-time PCR system. Likewise, the melting profiles are recorded automatically, stored electronically in a spreadsheet format and can be retrieved at any time point for comparative analyses. Because of its simplicity, HRM analysis offers a cost-effective yet accurate alternative to other probe-based genotyping assays such as SSCP, RFLP and DNA sequencing. This approach could be applicable to a wide range of microorganisms of medical importance especially closely related species diagnosed in a clinical laboratory.

## Supporting Information

Figure S1 **Calculation of the sensitivity and specificity for both conventional semi-nested PCR and HRM-real-time PCR assay.**
(DOC)

Figure S2 **The HRM profile, i.e., normalized fluorescence curves (above) and derivative melt curve (below) of nine out of 11 samples in which hookworm-like eggs were seen via microscopy however failed to be amplified in our conventional PCR.** These samples were amplified and identified as *N. americanus* based on their melting profile in HRM assay.
(TIF)
