# Supplementary material for: Rapid Detection and Identification of Human Hookworm Infections through High Resolution Melting (HRM) Analysis
Source: PLoS One. 2012 Jul 26;7(7):e41996. doi: 10.1371/journal.pone.0041996 (PMC3406038; doi:10.1371/journal.pone.0041996)
Supplement: Figure S1 — Calculation of the sensitivity and specificity for both conventional semi-nested PCR and HRM-real-time PCR assay. (DOC) [file pone.0041996.s001.doc]

**Figure S1**

**Sensitivity and specificity of the conventional semi-nested PCR and HRM-real-time PCR assay**

**1. Sensitivity and specificity of conventional semi-nested PCR**

True Positive=58; True Negative =576; False Positive=0; False Negative=11

Sensitivity = True positive ÷ (True positive + False negative) × 100

= 58 ÷ (58 + 11) × 100

= 84.1%

Specificity = True negative ÷ (True negative + False positive) × 100

= 576 ÷ (576 + 0) × 100

= 100%

**2. Sensitivity and specificity of HRM-real-time PCR assay**

True Positive=58; True Negative =576; False Positive=0; False Negative=0

Sensitivity = True positive ÷ (True positive + False negative) × 100

= 58 ÷ (58 + 0) × 100

= 100%

Specificity = True negative ÷ (True negative + False positive) × 100

= 576 ÷ (576 + 0) × 100

= 100%
