# Supplementary figures and images for: Rapid Detection and Identification of Human Hookworm Infections through High Resolution Melting (HRM) Analysis
Source: PLoS One. 2012 Jul 26;7(7):e41996. doi: 10.1371/journal.pone.0041996 (PMC3406038; doi:10.1371/journal.pone.0041996)

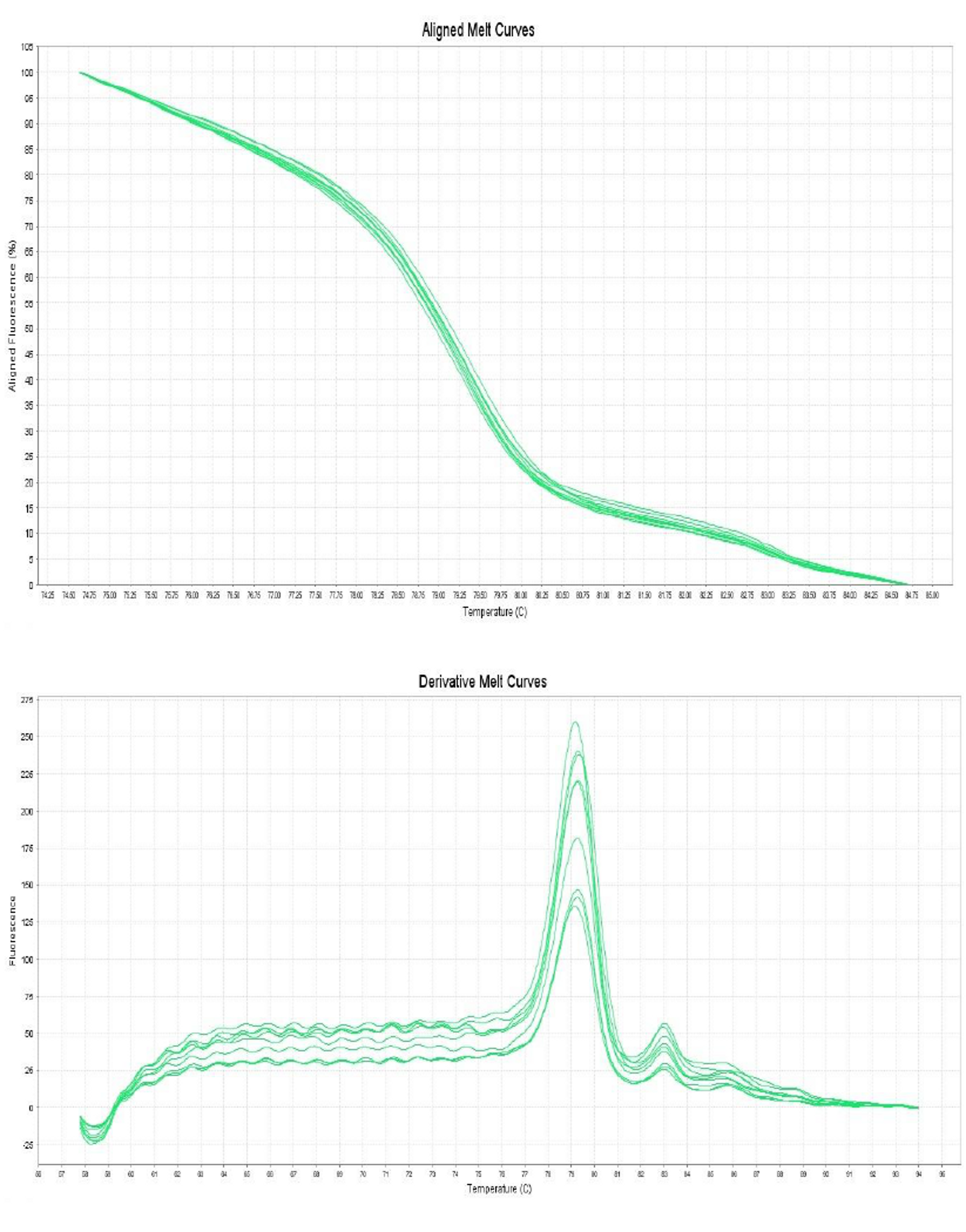

Supplement: Figure S2 — The HRM profile, i.e., normalized fluorescence curves (above) and derivative melt curve (below) of nine out of 11 samples in which hookworm-like eggs were seen via microscopy however failed to be amplified in our conventional PCR. These samples were amplified and identified as N. americanus based on their melting profile in HRM assay. (TIF) [file pone.0041996.s002.tif]
